# Supplementary material for: Thromboelastometry-Guided Individualized Fibrinolytic Treatment for COVID-19-Associated Severe Coagulopathy Complicated by Portal Vein Thrombosis: A Case Report
Source: Biomedicines. 2023 Sep 5;11(9):2463. doi: 10.3390/biomedicines11092463 (PMC10525483; doi:10.3390/biomedicines11092463)
Supplement: Supplementary file 1 [file biomedicines-11-02463-s001.zip › biomedicines-2525786-supplementary.pdf]

Supplementary material

Figure S1. – Effects of the adjuvant hemoadsoprtion treatment

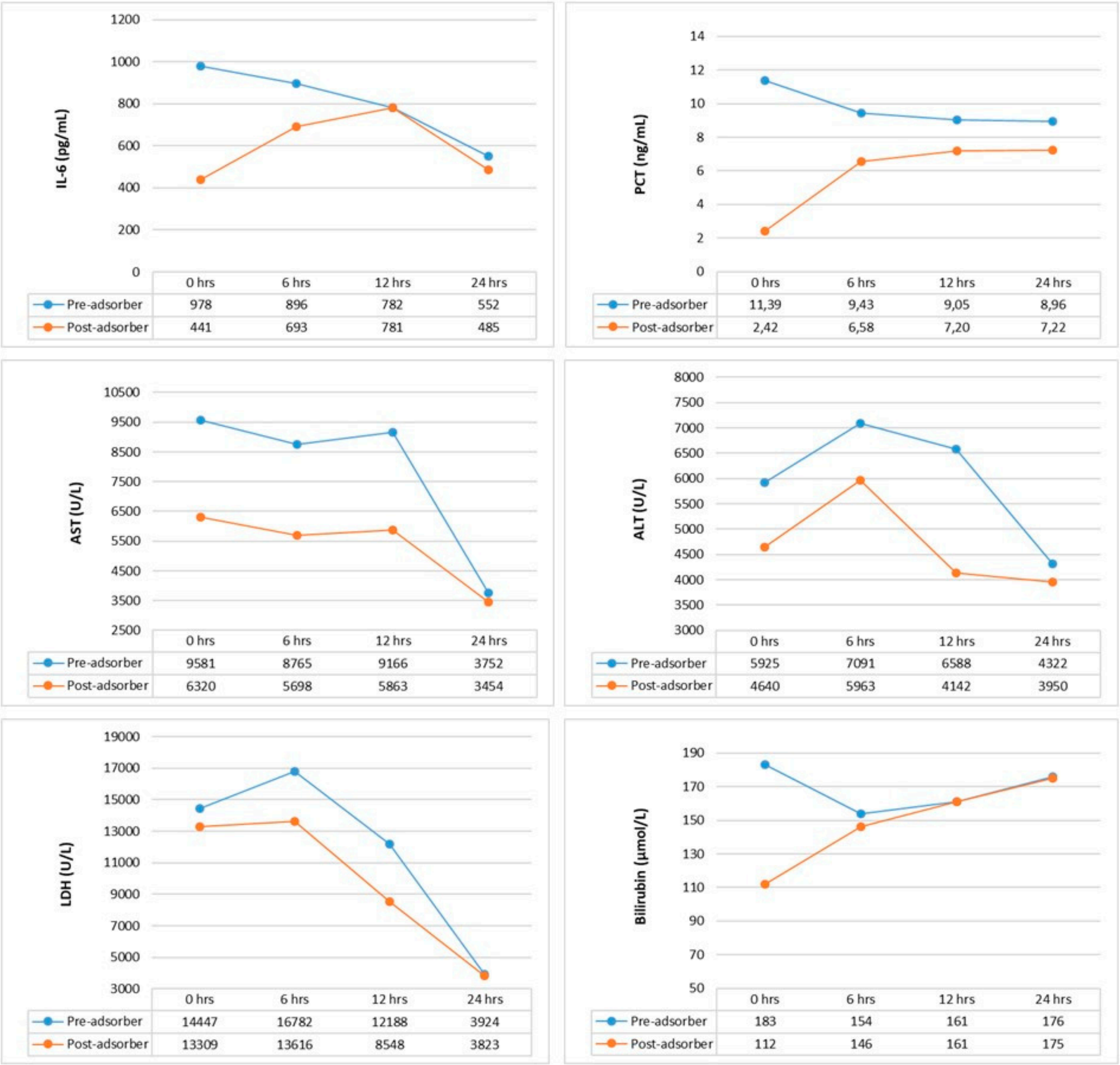

To evaluate the efficacy and the adsorption profile of the CytoSorb device (CytoSorbents Europe GmbH, Berlin, Germany) we collected blood samples from the hemodialysis circuit immediately before and after the adsorber 6 hourly to determine the removal of inflammatory markers (interleukin-6, procalcitonin), bilirubin and transaminases (AST, ALT, LDH). At the beginning (0 hrs) obvious removal was observed regarding all measured parameters. In general, serum concentrations also decreased during the treatment. However, a marked decrease occurred only after 12 hours, indicating a significant attenuation in systemic inflammation, while the narrowing gap between the pre-, and post-adsorber values indicated the decline in adsorption capacity and efficiency suggesting the saturation of the cartridge.

Abbreviations: IL-6, interleukin-6, PCT, procalcitonin, AST, aspartate aminotransferase; ALT, alanine aminotransferase; LDH, lactate dehydrogenase
